# Measurement of Stokes-operator squeezing for continuous-variable orbital angular momentum

**DOI:** 10.1038/s41598-017-04713-6

**Published:** 2017-06-30

**Authors:** Jun Guo, Chunxiao Cai, Long Ma, Kui Liu, Hengxin Sun, Jiangrui Gao

**Affiliations:** 1 State Key Laboratory of Quantum Optics and Quantum Optics Devices, Institute of Opto-Electronics, Shanxi University, Taiyuan, 030006 China; 2 Collaborative Innovation Center of Extreme Optics, Shanxi University, Taiyuan, Shanxi 030006 People’s Republic of China

## Abstract

We demonstrate experimentally a measurement scheme for the Stokes operators for the continuous-variable squeezed states of orbital angular momentum (OAM). An OAM squeezed state is generated by coupling a dim Hermite-Gauss HG_01_-mode quadrature-squeezed light beam with a bright HG_10_-mode coherent light beam on a 98/2 beam splitter. Using an asymmetric Mach–Zehnder interferometer with an extra Dove prism in one arm, we measured the three orbital Stokes operators of the OAM squeezed states with a self-homodyne detection and finally characterized their positions and noise on the orbital Poincaré sphere.

## Introduction

Propagating light beams carry spin angular momenta (SAM) associated with the polarizations and orbital angular momenta (OAM) related to the spatial helical phase structures^1^. Recently, the quantum OAM states have attracted increasing attention because of added increases in dimensionality of the associated Hilbert space and their potentials for quantum imaging^2^, quantum metrology^3^, and quantum storage^4^. For example, the quantum OAM states can be applied to measure the rotation angle of optical beams beyond the shot-noise limit (SNL)^5^. Another important application of the OAM states is their connectivity with atoms, allowing for storage of quantum information^6, 7^.

Compared with the vast majority of research on discrete OAM states in the single-photon regime^8–10^, there has been very little work concerning the continuous-variable (CV) OAM states. CV entanglement between two Laguerre-Gauss modes was first realized in a hot vapour based on four-wave mixing^11, 12^. CV entanglement between the first-order OAM states has also been produced in a type-I optical parametric oscillator (OPO) and the orbital Stokes operators of the OAM states were demonstrated to be squeezed^13^. CV hyper-entanglement, i.e., simultaneous entanglements of SAM and OAM, has been theoretically predicted^14^ and experimentally realized in a multimode type-II OPO^15^.

However, in refs 13 and 15, the squeezing and entanglement of the orbital Stokes operators were inferred from the measured quadrature squeezing and entanglement of Hermite–Gauss and Laguerre–Gauss modes based on balanced homodyne detection with spatially tailored local oscillators. In 2009, Lam *et al*. theoretically proposed a spatial detection scheme comprised of an asymmetric Mach–Zehnder interferometer and a pair of cylindrical lenses to measure all three Stokes operators of OAM^16^, but until now no experiment has been reported.

In this Letter, we propose and demonstrate experimentally a scheme to measure all three Stokes operators of OAM requiring an asymmetric Mach–Zehnder interferometer with a Dove prism in one arm. This scheme is more convenient to operate in experiments, and the set-up is broadly applicable to the first-order OAM states. With no local oscillator needed, the first-order OAM states entering the set-up can be measured. In contrast, for balanced homodyne detection, a local oscillator is required that is spatially tailored to the first-order mode to be measured. In addition, our scheme is more efficient in certain nonlocal quantum information protocols, in which it is hard to select the optimal local oscillators, such as quantum state transmission^17^ and quantum key distribution^18^.

## Orbital angular momentum squeezed state

**Figure 1:**
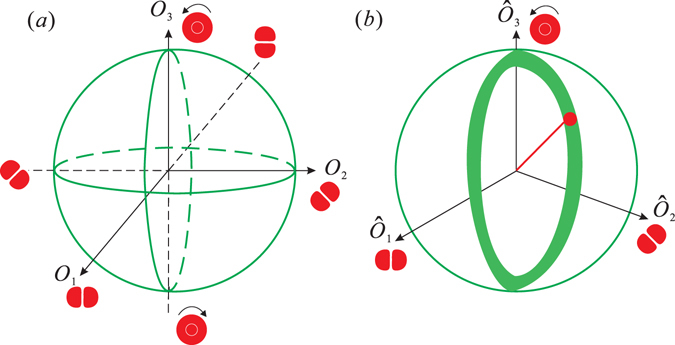
(**a**) Orbital Poincaré sphere for the first-order spatial modes. (**b**) Quantum noise representation of OAM state (thick ring) on the Poincaré sphere.

Similar to the polarization of light, the first-order spatial modes can also be characterized by the orbital Stokes operators and mapped onto the orbital Poincaré sphere^16, 19^. Such a sphere is displayed in Fig. 1 for the first-order OAM modes.

The quantum orbital Stokes operators for the first-order OAM modes can be expressed as^16^

1 $$\begin{array}{rcl}{\hat{O}}_{0} & = & {\hat{a}}_{10}^{\dagger }{\hat{a}}_{10}+{\hat{a}}_{01}^{\dagger }{\hat{a}}_{01}\\ {\hat{O}}_{1} & = & {\hat{a}}_{10}^{\dagger }{\hat{a}}_{10}-{\hat{a}}_{01}^{\dagger }{\hat{a}}_{01}\\ {\hat{O}}_{2} & = & {\hat{a}}_{10}^{\dagger }{\hat{a}}_{01}{e}^{i\phi }+{\hat{a}}_{01}^{\dagger }{\hat{a}}_{10}{e}^{-i\phi }\\ {\hat{O}}_{3} & = & i{\hat{a}}_{01}^{\dagger }{\hat{a}}_{10}{e}^{-i\phi }-i{\hat{a}}_{10}^{\dagger }{\hat{a}}_{01}{e}^{i\phi },\end{array}$$

where ${\hat{O}}_{0}$, ${\hat{O}}_{1}$, ${\hat{O}}_{2}$ and ${\hat{O}}_{3}$ denote respectively operators corresponding to the total number of photons, the difference in photon number between modes HG_10_ and HG_01_ modes, and likewise between the pair of modes $H{G}_{10}^{{45}^{\circ }}$ and $H{G}_{10}^{{135}^{\circ }}$, and modes $L{G}_{0}^{+1}$ and $L{G}_{0}^{-1}$ ^13, 15^. ${\hat{a}}_{10(01)}^{\dagger }$ and ${\hat{a}}_{10(01)}$ are the creation and annihilation operators for the HG_10_ (HG_01_) modes, *φ* is the phase difference between modes HG_10_ and HG_01_. The definitions of ${\hat{O}}_{2}$ and ${\hat{O}}_{3}$ in Eq. (1) contain the product of annihilation operators relative to two different field modes. This in turn implies that to have an apparatus able to effectively realise this operator, spatial and temporal profiles of the two modes have to match optimally (perfectly in the ideal case) and a control on the relative phase between the two modes is needed.

The annihilation operators of photons can be linearized as ${\hat{a}}_{10(01)}={\alpha }_{10(01)}+{\rm{\Delta }}{\hat{a}}_{10(01)}$, where ${\alpha }_{{\rm{10}}({\rm{01}})}$ represents the mean amplitude and ${\rm{\Delta }}{\hat{a}}_{10(01)}$ is the quantum noise operator. Introducing the amplitude and phase quadrature operators $\hat{X}=\hat{a}+{\hat{a}}^{\dagger }$ and $\hat{Y}=-i(\hat{a}-{\hat{a}}^{\dagger })$, then

2 $${\rm{\Delta }}{\hat{a}}_{\mathrm{10(01)}}=\frac{1}{2}({\rm{\Delta }}{\hat{X}}_{\mathrm{10(01)}}+i{\rm{\Delta }}{\hat{Y}}_{\mathrm{10(01)}})\mathrm{.}$$

The noise variances of the Stokes operators shown in Fig. 1(b) are obtained as

3 $$\begin{array}{rcl}{{\rm{\Delta }}}^{2}{\hat{O}}_{0} & = & {\alpha }_{10}^{2}{{\rm{\Delta }}}^{2}{\hat{X}}_{10}+{\alpha }_{01}^{2}{{\rm{\Delta }}}^{2}{\hat{X}}_{01}\\ {{\rm{\Delta }}}^{2}{\hat{O}}_{1} & = & {\alpha }_{10}^{2}{{\rm{\Delta }}}^{2}{\hat{X}}_{10}+{\alpha }_{01}^{2}{{\rm{\Delta }}}^{2}{\hat{X}}_{01}\\ {{\rm{\Delta }}}^{2}{\hat{O}}_{2} & = & {\cos }^{2}\phi ({\alpha }_{01}^{2}{{\rm{\Delta }}}^{2}{\hat{X}}_{10}+{\alpha }_{10}^{2}{{\rm{\Delta }}}^{2}{\hat{X}}_{01})+{\sin }^{2}\phi ({\alpha }_{01}^{2}{{\rm{\Delta }}}^{2}{\hat{Y}}_{10}+{\alpha }_{10}^{2}{{\rm{\Delta }}}^{2}{\hat{Y}}_{01})\\ {{\rm{\Delta }}}^{2}{\hat{O}}_{3} & = & {\sin }^{2}\phi ({\alpha }_{01}^{2}{{\rm{\Delta }}}^{2}{\hat{X}}_{10}+{\alpha }_{10}^{2}{\Delta }^{2}{\hat{X}}_{01})+{\cos }^{2}\phi ({\alpha }_{01}^{2}{{\rm{\Delta }}}^{2}{\hat{Y}}_{10}+{\alpha }_{10}^{2}{{\rm{\Delta }}}^{2}{\hat{Y}}_{01}).\end{array}$$

**Figure 2:**
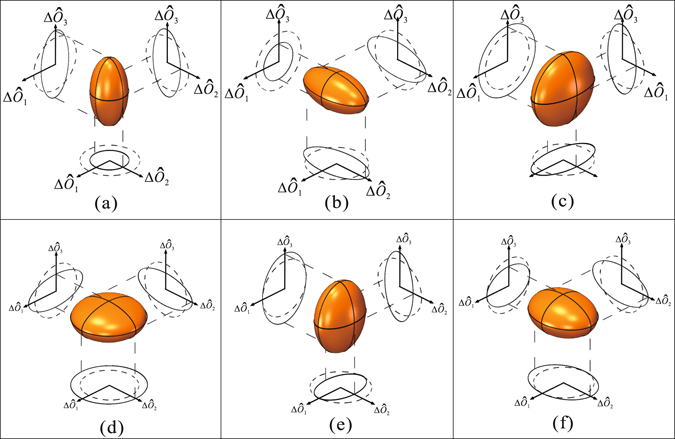
Orbital Poincaré spheres for the different types of OAM squeezed states.

Here ${{\rm{\Delta }}}^{2}{\hat{X}}_{10(01)}$ and ${{\rm{\Delta }}}^{2}{\hat{Y}}_{10(01)}$ are the noise variances of amplitude quadrature ($\hat{X}=\hat{a}+{\hat{a}}^{\dagger }$) and phase quadrature ($\hat{Y}=-i(\hat{a}-{\hat{a}}^{\dagger })$) operators for ${\hat{a}}_{10(01)}$ modes. ${\alpha }_{10(01)}$ are the mean amplitudes for the two modes. These equations state that different types of OAM squeezed states can be generated through combinations of quadrature-squeezed states. For example, if we couple two amplitude-squeezed states of the HG_10_ and HG_01_ modes [i.e., ${{\rm{\Delta }}}^{2}{\hat{X}}_{01} < 1$ and ${{\rm{\Delta }}}^{2}{\hat{X}}_{10} < 1$] with their relative phase *φ* = 0, then squeezing of the operators ${\hat{O}}_{0},{\hat{O}}_{1},{\hat{O}}_{2}$ is obtained; ${\hat{O}}_{3}$ appears anti-squeezed when mapped onto the orbital Poincaré sphere. The volume of quantum noise is represented as a “cigar-like” ellipsoid [Fig. 2(a)], which has been generated in ref. 13. When *φ* = *π*/2, then squeezing of ${\hat{O}}_{0},{\hat{O}}_{1},{\hat{O}}_{3}$ is achieved, and ${\hat{O}}_{2}$ is anti-squeezed. The state is also cigar-like [Fig. 2(b)]. If we couple two phase-squeezed states for modes HG_10_ and HG_01_ [i.e., ${{\rm{\Delta }}}^{2}{\hat{Y}}_{01} < 1$ and ${{\rm{\Delta }}}^{2}{\hat{Y}}_{10} < 1$] with their relative phase *φ* = 0, then ${\hat{O}}_{0}$, ${\hat{O}}_{1}$, ${\hat{O}}_{2}$ are anti-squeezed and only ${\hat{O}}_{3}$ is squeezed when mapped onto the Poincaré sphere; a “pancake-like” ellipsoid is produced [Fig. 2(d)]. When *φ* = *π*/2, only ${\hat{O}}_{2}$ is squeezed, whereas ${\hat{O}}_{0}$, ${\hat{O}}_{1}$, ${\hat{O}}_{3}$ are anti-squeezed. The state is also pancake-like [Fig. 2(c)]. In addition, if we couple a bright coherent HG_10_ mode [i.e., ${{\rm{\Delta }}}^{2}{\hat{X}}_{10}=1$] with a dim amplitude squeezed HG_01_ mode [i.e., ${{\rm{\Delta }}}^{2}{\hat{X}}_{01} < 1$] or a bright coherent HG_01_ mode with a dim amplitude squeezed HG_10_ mode with *φ* = 0, then ${\hat{O}}_{2}$ is squeezed, ${\hat{O}}_{3}$ is anti-squeezed, and ${\hat{O}}_{0}$ and ${\hat{O}}_{1}$ are shot noise limited (SNL). The quantum spheres are “pancake-like” ellipsoids [Fig. 2(e)]. If *φ* = *π*/2, ${\hat{O}}_{3}$ is squeezed, ${\hat{O}}_{2}$ is anti-squeezed, and ${\hat{O}}_{0}$ and ${\hat{O}}_{1}$ are SNL. The state is also pancake-like [Fig. 2(f)]. Here the quantum states shown in Fig. 2(e) and (f) are generated and measured in the experiment.

## Detection scheme

**Figure 3:**
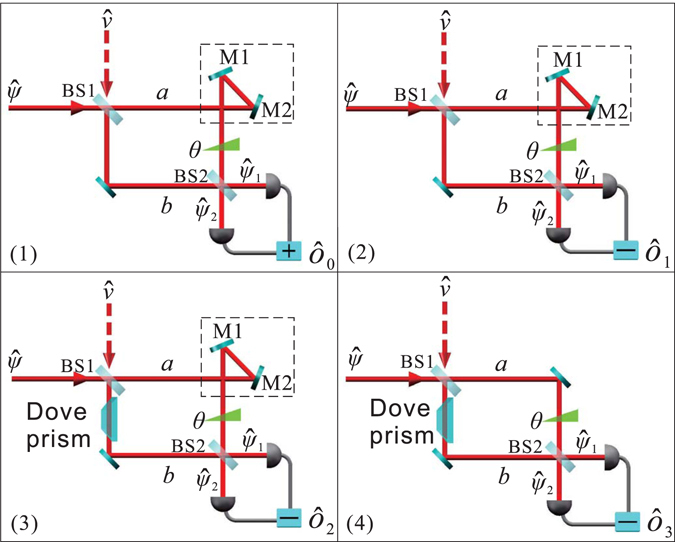
Detection scheme for the four orbital Stokes operators ${\hat{O}}_{0},{\hat{O}}_{1},{\hat{O}}_{2},{\hat{O}}_{3}$.

To measure the three orbital Stokes operators (see Fig. 3), we propose a scheme based on the asymmetric Mach–Zehnder interferometer with a Dove prism in one arm. The Dove prism is used to convert a HG_10_ mode into a HG_01_ mode or vice versa. In an asymmetric Mach–Zehnder interferometer, there are two mirrors M1 and M2 in arm a^16, 20^ that add an extra phase *e* ^*iπ*^ to the HG_10_ mode but have no effect on the HG_01_ mode. M1 and M2 are the same for the first three schemes (1), (2), and (3) used in the detection of ${\hat{O}}_{0},{\hat{O}}_{1},{\hat{O}}_{2}$. In scheme (4) for ${\hat{O}}_{3}$, unlike the first three schemes, there is only a single mirror in *a* arm, and hence creates a symmetric Mach–Zehnder interferometer having a Dove prism in *b* arm.

Any first order spatial mode *ψ* can be expressed as

4 $$\hat{\psi }={\hat{a}}_{01}{u}_{01}(\vec{r})+{e}^{i\phi }{\hat{a}}_{10}{u}_{10}(\vec{r}),$$

where ${u}_{01(10)}(\vec{r})$ are the normalized transverse beam amplitude functions for modes HG_01_ (HG_10_), and *φ* is the phase difference between modes HG_10_ and HG_01_. An account of the quantum vacuum noise entering the setup through the unused port BS1 is introduced by defining the operator

5 $$\hat{\nu }={\hat{a}}_{01}^{\nu }{u}_{01}(\vec{r})+{e}^{i\phi }{\hat{a}}_{10}^{\nu }{u}_{10}(\vec{r}),$$

where ${\hat{a}}_{01(10)}^{\nu }$ are the quantum vacuum noise operators for HG_01_ (HG_10_) modes.

With the presence of the two mirrors M1 and M2, in *a* arm for schemes (1) and (2), the HG_10_ mode receives an extra phase *e* ^*iπ*^, that is, *u*_10_ → −*u*_10_, while HG_01_ mode is not changed, *u*_01_ → *u*_01_; hence,

6 $${\hat{\psi }}_{a}=\frac{1}{\sqrt{2}}({\hat{a}}_{01}{u}_{01}-{e}^{i\phi }{\hat{a}}_{10}{u}_{10}+{\hat{a}}_{01}^{\nu }{u}_{01}-{e}^{i\phi }{\hat{a}}_{10}^{\nu }{u}_{10}),$$

whereas in *b* arm, we have

7 $${\hat{\psi }}_{b}=\frac{1}{\sqrt{2}}({\hat{a}}_{01}{u}_{01}+{e}^{i\phi }{\hat{a}}_{10}{u}_{10}-{\hat{a}}_{01}^{\nu }{u}_{01}-{e}^{i\phi }{\hat{a}}_{10}^{\nu }{u}_{10}),$$

The two output states of beam splitter BS2 are

8 $$\begin{array}{rcl}{\hat{\psi }}_{1} & = & \frac{1}{\sqrt{2}}({\hat{\psi }}_{a}+{e}^{i\theta }{\hat{\psi }}_{b})\\ {\hat{\psi }}_{2} & = & \frac{1}{\sqrt{2}}({\hat{\psi }}_{a}-{e}^{i\theta }{\hat{\psi }}_{b}),\end{array}$$

where *θ* is the relative phase between the two arms of the interferometer. When *θ* = 0, the sum of the two photocurrents is

9 $$\int {\hat{\psi }}_{1}^{\dagger }{\hat{\psi }}_{1}d{\vec{r}}^{2}+\int {\hat{\psi }}_{2}^{\dagger }{\hat{\psi }}_{2}d{\vec{r}}^{2}={\hat{a}}_{01}^{\dagger }{\hat{a}}_{01}+{\hat{a}}_{10}^{\dagger }{\hat{a}}_{10}={\hat{O}}_{0},$$

which is just the first orbital Stokes operator as defined in Eq. (1). The difference in the two photocurrents is

10 $$\int {\hat{\psi }}_{2}^{\dagger }{\hat{\psi }}_{2}d{\vec{r}}^{2}-\int {\hat{\psi }}_{1}^{\dagger }{\hat{\psi }}_{1}d{\vec{r}}^{2}={\hat{a}}_{10}^{\dagger }{\hat{a}}_{10}-{\hat{a}}_{01}^{\dagger }{\hat{a}}_{01}={\hat{O}}_{1},$$

which is the second orbital Stokes operator [see Eq. (1)].

For *a* arm of scheme (3), the equation is the same as Eq. (5), whereas in *b* arm, the Dove prism is used to rotate the mode by 90°, that is, *u*_01_ → *u*_10_, *u*_10_ → −*u*_01_, and therefore

11 $${\hat{\psi }}_{b}=\frac{1}{\sqrt{2}}({\hat{a}}_{01}{u}_{10}-{e}^{i\phi }{\hat{a}}_{10}{u}_{01}-{\hat{a}}_{01}^{\nu }{u}_{10}+{e}^{i\phi }{\hat{a}}_{10}^{\nu }{u}_{01}),$$

when *θ* = 0. The difference between the two photocurrents is

12 $$\int {\hat{\psi }}_{2}^{\dagger }{\hat{\psi }}_{2}d{\vec{r}}^{2}-\int {\hat{\psi }}_{1}^{\dagger }{\hat{\psi }}_{1}d{\vec{r}}^{2}={\hat{a}}_{01}^{\dagger }{\hat{a}}_{10}{e}^{i\phi }+{\hat{a}}_{10}^{\dagger }{\hat{a}}_{01}{e}^{-i\phi }={\hat{O}}_{2},$$

which is the third orbital Stokes operator [see Eq. (1)].

For *a* arm of scheme (4),

13 $${\hat{\psi }}_{a}=\frac{1}{\sqrt{2}}({\hat{a}}_{01}{u}_{01}+{e}^{i\phi }{\hat{a}}_{10}{u}_{10}+{\hat{a}}_{01}^{\nu }{u}_{01}+{e}^{i\phi }{\hat{a}}_{10}^{\nu }{u}_{10}),$$

whereas in *b* arm, with the Dove prism inserted, the state is the same as Eq. (10). When $\theta =\frac{\pi }{2}$,

14 $$\int {\hat{\psi }}_{1}^{\dagger }{\hat{\psi }}_{1}d{\vec{r}}^{2}-\int {\hat{\psi }}_{2}^{\dagger }{\hat{\psi }}_{2}d{\vec{r}}^{2}=i{\hat{a}}_{10}^{\dagger }{\hat{a}}_{01}{e}^{-i\phi }-i{\hat{a}}_{01}^{\dagger }{\hat{a}}_{10}{e}^{i\phi }={\hat{O}}_{3}\mathrm{.}$$

which is the fourth orbital Stokes operator [see Eq. (1)]. Therefore, we can use the detection scheme to measure the four orbital Stokes operators^16^, and the quantum vacuum noise has no effect on the results.

Considering the imperfection of the setup and assuming the mode conversion efficiency *η*_1_ of the two mirrors M1 and M2 and *η*_2_ of the Dove prism, the modes through the conversions of mirrors M1 and M2 become ${u}_{10}\to -\sqrt{{\eta }_{1}}{u}_{10}+\sqrt{1-{\eta }_{1}}{u}_{01}$, ${u}_{01}\to \sqrt{{\eta }_{1}}{u}_{01}+\sqrt{1-{\eta }_{1}}{u}_{10}$, and the modes after the rotations of Dove prism become ${u}_{10}\to -\sqrt{{\eta }_{2}}{u}_{01}+\sqrt{1-{\eta }_{2}}{u}_{10}$, ${u}_{01}\to \sqrt{{\eta }_{2}}{u}_{10}+\sqrt{1-{\eta }_{2}}{u}_{01}$. Then the result for scheme (1) is still ${\hat{O}}_{0}$. For scheme (2), it becomes $\sqrt{{\eta }_{1}}{\hat{O}}_{1}+\sqrt{1-{\eta }_{1}}{\hat{O}}_{2}$, and the noise becomes ${\eta }_{1}{{\rm{\Delta }}}^{{\rm{2}}}{\hat{O}}_{1}+(1-{\eta }_{1}){{\rm{\Delta }}}^{{\rm{2}}}{\hat{O}}_{2}$. For scheme (3), it becomes $(\sqrt{{\eta }_{1}}\sqrt{1-{\eta }_{2}}+\sqrt{{\eta }_{2}}\sqrt{1-{\eta }_{1}}){\hat{O}}_{1}+(\sqrt{{\eta }_{1}}\sqrt{{\eta }_{2}}-\sqrt{1-{\eta }_{1}}\sqrt{1-{\eta }_{2}}){\hat{O}}_{2}$, neglecting the second order small terms $(1-{\eta }_{1})(1-{\eta }_{2})$ and $\sqrt{1-{\eta }_{1}}\sqrt{1-{\eta }_{2}}$, the noise becomes $[{\eta }_{1}(1-{\eta }_{2})+{\eta }_{2}(1-{\eta }_{1})]{{\rm{\Delta }}}^{2}{\hat{O}}_{1}+{\eta }_{1}{\eta }_{2}{{\rm{\Delta }}}^{2}{\hat{O}}_{2}$. For scheme (4), the result is $\sqrt{{\eta }_{2}}{\hat{O}}_{3}+\sqrt{1-{\eta }_{2}}(|{\alpha }_{01}|{\hat{Y}}_{01}+|{\alpha }_{10}|{\hat{Y}}_{10})$, and the noise becomes ${\eta }_{2}{{\rm{\Delta }}}^{2}{\hat{O}}_{3}+(1-{\eta }_{2})({|{\alpha }_{01}|}^{2}{{\rm{\Delta }}}^{2}{\hat{Y}}_{01}+{|{\alpha }_{10}|}^{2}{{\rm{\Delta }}}^{2}{\hat{Y}}_{10})$. Therefore, the imperfections will cause the coupling of different Stokes operators, degrading the detection accuracies for the three Stokes operators. On the other hand, considering the mode matching efficiency *ξ* ^2^ between the two arms caused by misalignment of Mach–Zehnder interferometer, the optical transmission efficiency *η*_*tr*_ and the quantum efficiency of photodetectors *η*_*phot*_, the noise of ${\hat{O}}_{1}$ becomes ${\eta }_{1}{\eta }_{{\rm{\det }}}{{\rm{\Delta }}}^{{\rm{2}}}{\hat{O}}_{1}+(1-{\eta }_{1}){\eta }_{{\rm{\det }}}{{\rm{\Delta }}}^{{\rm{2}}}{\hat{O}}_{2}+1-{\eta }_{{\rm{\det }}}$, where ${\eta }_{{\rm{\det }}}={\eta }_{tr}{\eta }_{phot}{\xi }^{2}$. Similarly, the noise of ${\hat{O}}_{2}$ becomes $[{\eta }_{1}(1-{\eta }_{2})+{\eta }_{2}(1-{\eta }_{1})]{\eta }_{{\rm{\det }}}{{\rm{\Delta }}}^{2}{\hat{O}}_{1}+{\eta }_{1}{\eta }_{2}{\eta }_{{\rm{\det }}}{{\rm{\Delta }}}^{2}{\hat{O}}_{2}+1-{\eta }_{{\rm{\det }}}$, and the noise of ${\hat{O}}_{3}$ becomes ${\eta }_{2}{\eta }_{{\rm{\det }}}{{\rm{\Delta }}}^{2}{\hat{O}}_{3}+(1-{\eta }_{2}){\eta }_{{\rm{\det }}}({|{\alpha }_{01}|}^{2}{{\rm{\Delta }}}^{2}{\hat{Y}}_{01}+{|{\alpha }_{10}|}^{2}{{\rm{\Delta }}}^{2}{\hat{Y}}_{10})+1-{\eta }_{{\rm{\det }}}$, so all these inefficiencies will introduce the vacuum noise and degrade the detection efficiencies for ${\hat{O}}_{1}$, ${\hat{O}}_{2}$ and ${\hat{O}}_{3}$, further degrading the degree of the measured squeezing in experiment.

## Experimental set-up

**Figure 4:**
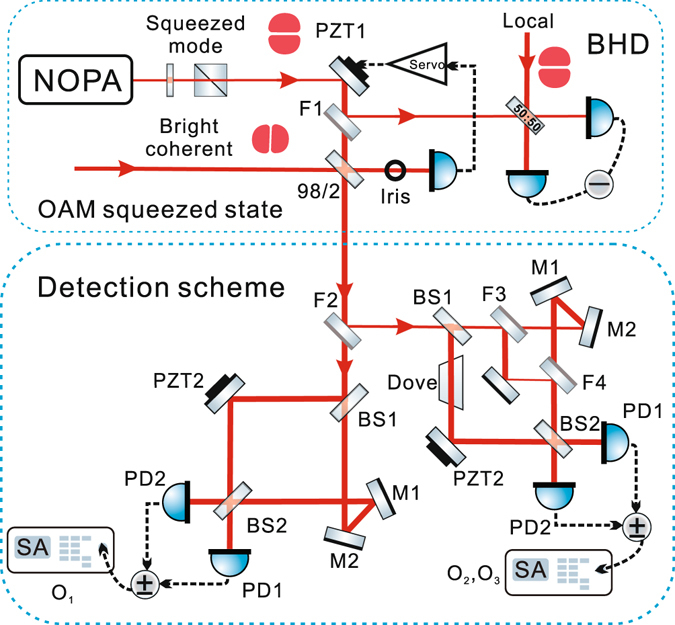
Experimental set-up for the generation and detection of OAM squeezed states. PZTs: piezoelectric transducers, 98/2: 98/2 beam splitter, BSs: 50/50 beam splitter, Ms: high-reflectivity mirrors, Fs: flip mirrors, Dove: Dove prism, PDs: photodetectors,(+/−): positive/negative combiner, SA: spectrum analyser, Servo: servo amplifier circuit for feedback system.

Referring to the experimental set-up illustrated in Fig. 4, a two mode squeezed state of 1080 nm for HG_01_ mode is generated from a NOPA^15, 21^, then the bright mode which is an amplitude squeezed state is separated by the combination of a half wave plate and a polarizing beam splitter^22^. The HG_01_ mode squeezed state is firstly detected by a balanced homodyne detection with the flip mirror F1 on, and the squeezing value is obtained. Then F1 is turned off, the HG_01_ squeezed state with power of 30 *μW* is coupled with a bright coherent HG_10_ mode of 1080 nm with power of 100 mW on a 98/2 beam splitter, ensuring the squeezing power of 98% is transmitted and the coherent power of 2% is reflected, generating the OAM squeezed state. As the definition in Eq. (1), the coupling of HG_01_ mode and HG_10_ mode requires mode matching and phase locking, since the HG_01_ mode and the HG_10_ mode are two orthogonal modes, we assess the mode matching by the interference between the HG_00_ modes which are eigenmodes orthogonal with the HG_10_ modes on the 98/2 beam splitter. In addition, we use an iris to acquire part of the interference of HG_01_ and HG_10_ modes to control the relative phase through a servo system and PZT1, generating different types of OAM squeezed states. We lock the relative phase *φ* to 0 or $\frac{\pi }{2}$ in the experiment.

The OAM squeezed states are measured implementing the scheme discussed in the previous section (see Fig. 3). As shown in Fig. 4, we use a flip mirror (F2) to choose where the OAM squeezed state goes. If F2 is off/on, the state goes to the detection scheme for ${\hat{O}}_{1}$/${\hat{O}}_{2}$ and ${\hat{O}}_{3}$. The other two flip mirrors, F3 and F4, are used to determine whether ${\hat{O}}_{2}$ or ${\hat{O}}_{3}$ is detected; if F3 and F4 are both off/on, the asymmetric/symmetric Mach–Zehnder interferometer is active, and hence ${\hat{O}}_{2}$/${\hat{O}}_{3}$ is detected. PZT2 and PZT3 are used to lock the relative phases *θ* between the two arms of the Mach–Zehnder interferometers. When ${\hat{O}}_{1}$ and ${\hat{O}}_{2}$ are being measured, *θ* is locked to zero; when ${\hat{O}}_{3}$ is being measured, *θ* is locked to $\frac{\pi }{2}$. The two outputs of the interferometers enter two photodetectors, and the photocurrents feed a positive/negative combiner (+/−), and these outputs are recorded by a spectrum analyser (SA). A positive combiner (+) determines the SNL; a negative combiner (−) determines the noise of the orbital Stokes operators.

In our scheme, a local oscillator is unneeded, so it is more efficient in certain nonlocal quantum information protocols, such as free-space quantum state distribution^17, 18^ which has the potential to form a key component in future quantum networks. In the squeezing enhanced CV quantum key distribution (QKD) protocols, the decoherence as a result of phase relation variations and wave front distortions plays an important role in the degradation of the quantum states, thus standard homodyne measurements at the receiver are challenging^17^. Similar to polarization squeezed state, the OAM squeezed state based on our measurement scheme of Stokes operators is promising to supply a way to avoid the problem. Moreover, it can be expanded to high-dimensional CV QKD based on high-dimensional OAM.

## Experimental Results

**Figure 5:**
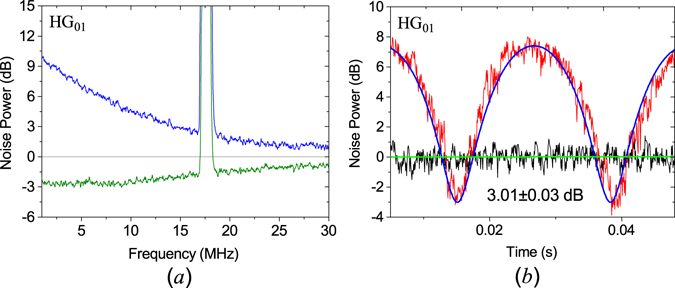
Noise power for the HG_01_ mode. (**a**) the squeezing and anti-squeezing values for HG_01_ mode from 1 MHz to 30 MHz. (**b**) the noise power for HG_01_ mode at 5 MHz.

Figure 5 gives the squeezing curves for HG_01_ mode, (a) is the squeezing and anti-squeezing values for HG_01_ mode from 1 MHz to 30 MHz. The squeezing exists over a large frequency domain of 1–30 MHz; (b) is the noise power for HG_01_ mode at 5 MHz. The squeezing value is −3.01 ± 0.03 dB at 5 MHz. Considering the overlap efficiency in balanced homodyne detection *η*_*hd*_ = 0.93 ± 0.01, and the quantum efficiency of the photodiode *η*_*phot*_ = 0.90 ± 0.02, the inferred squeezing is −3.95 ± 0.12 dB.

**Figure 6:**
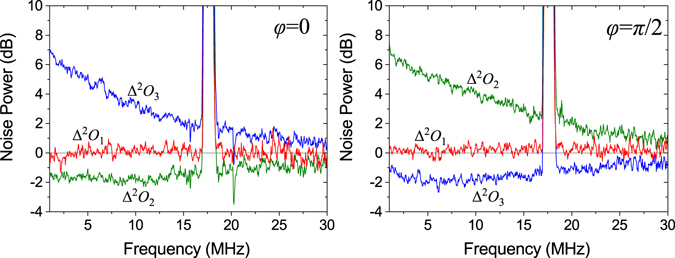
Noise power for the three orbital Stokes operators for OAM squeezed states with *φ* = 0 and *φ* = *π*/2.

The measured noise powers for the OAM Stokes operators are depicted in Fig. 6. The quantum noise for the first Stokes operator ${\hat{O}}_{1}$ is almost shot noise limited over the frequency domain 1–30 MHz for both *φ* = 0 and *φ* = *π*/2. At 5 MHz, the quantum noise for ${\hat{O}}_{1}$ is −0.07 ± 0.25 dB for *φ* = 0 and 0.12 ± 0.22 dB for *φ* = *π*/2. When *φ* = 0, the quantum noise for the second Stokes operator ${\hat{O}}_{2}$ is squeezed. The squeezing exists over a large frequency domain of 1–30 MHz; the squeezing of −1.70 ± 0.15 dB at 5 MHz is obtained. The third Stokes operator ${\hat{O}}_{3}$ is anti-squeezed, the anti-squeezing noise is 5.06 ± 0.06 dB at 5 MHz. When *φ* = *π*/2, the quantum noise for the third Stokes operator ${\hat{O}}_{3}$ is squeezed,and the squeezing also exists over a large frequency domain of 1–30 MHz, and the squeezing of–1.96 ± 0.16 dB at 5 MHz is obtained. The second Stokes operator ${\hat{O}}_{2}$ is anti-squeezed, the anti-squeezing noise is 5.06 ± 0.03 dB at 5 MHz. The peak at 18 MHz is a modulation signal for phase locking.

In experiment, the maximum coupling efficiency on the 98/2 beam splitter for HG_01_ and HG_10_ modes is *η*_*coup*_ = 0.93 ± 0.02, which is the mode matching efficiency of the HG_00_ modes, and considering the loss of 2% of the squeezed state, the total efficiency is *η*_*tot*_ = 0.91 ± 0.02. After the 98/2 beam splitter, the squeezing of the Stokes operators ${\hat{O}}_{2}$ and ${\hat{O}}_{3}$ should be −3.41 ± 0.2 dB at 5 MHz, which is inferred from the squeezing of −3.95 ± 0.12 dB for HG_01_ mode.

Considering the detection efficiency, for ${\hat{O}}_{2}$, the mode matching efficiency of the MZ interferometer is *ξ* ^2^ = 0.92 ± 0.02, the optical transmission efficiency is *η*_*tr*_ = 0.90 ± 0.02, and the quantum efficiency of photodetectors is *η*_*phot*_ = 0.90 ± 0.02, then the detection efficiency is *η*_det_ = *η*_*tr*_ *η*_*phot*_ *ξ* ^2^ = 0.75 ± 0.05, additionally considering the mode conversion efficiency *η*_1_ = 0.97 ± 0.01 of the two mirrors M1 and M2 and *η*_2_ = 0.97 ± 0.01 of the Dove prism, then the inferred noise of ${\hat{O}}_{2}$ at 5 MHz is −2.09 ± 0.36 dB when *φ* = *0*. For ${\hat{O}}_{3}$, the mode matching efficiency of the MZ interferometer is *ξ* ^2^ = 0.96 ± 0.02, the optical transmission efficiency is *η*_*tr*_ = 0.91 ± 0.02, and the quantum efficiency of photodetectors is *η*_*phot*_ = 0.90 ± 0.02, then the detection efficiency is *η*_det_ = *η*_*tr*_ *η*_*phot*_ *ξ* ^2^ = 0.79 ± 0.05, additionally considering *η*_2_ = 0.97 ± 0.01 of the Dove prism, then the inferred noise of ${\hat{O}}_{3}$ at 5 MHz is −2.33 ± 0.37 dB when *φ* = *π*/2. In addition, for ${\hat{O}}_{1}$, the mode matching efficiency is *ξ* ^2^ = 0.98 ± 0.02, the transmission efficiency is *η*_*tr*_ = 0.93 ± 0.02, and the quantum efficiency of photodetectors is *η*_*phot*_ = 0.90 ± 0.02, then the detection efficiency is *η*_det_ = *η*_*tr*_ *η*_*phot*_ *ξ* ^2^ = 0.82 ± 0.05, additionally considering *η*_1_ = 0.97 ± 0.01 of the two mirrors M1 and M2, when *φ* = 0, the inferred noise of ${\hat{O}}_{1}$ at 5 MHz is −0.08 ± 0.25 dB, when *φ* = *π*/2, the inferred noise of ${\hat{O}}_{1}$ at 5 MHz is 0.21 ± 0.25 dB.

Here, we use the HG_00_ mode interference to estimate the mode matching efficiency of the 98/2 beam splitter, the practical coupling efficiency for HG_01_ and HG_10_ modes on the 98/2 beam splitter should be lower than the estimate value, so the measured squeezing for ${\hat{O}}_{2}$ and ${\hat{O}}_{3}$ in experiment are lower than the inferred squeezing values. But for balanced homodyne detection^13, 15^, the measurement results of squeezing are independent of the mode matching efficiency between HG_01_ and HG_10_ modes on the 98/2 beam splitter, and it can’t infer accuracy results for Stokes operators.

**Figure 7:**
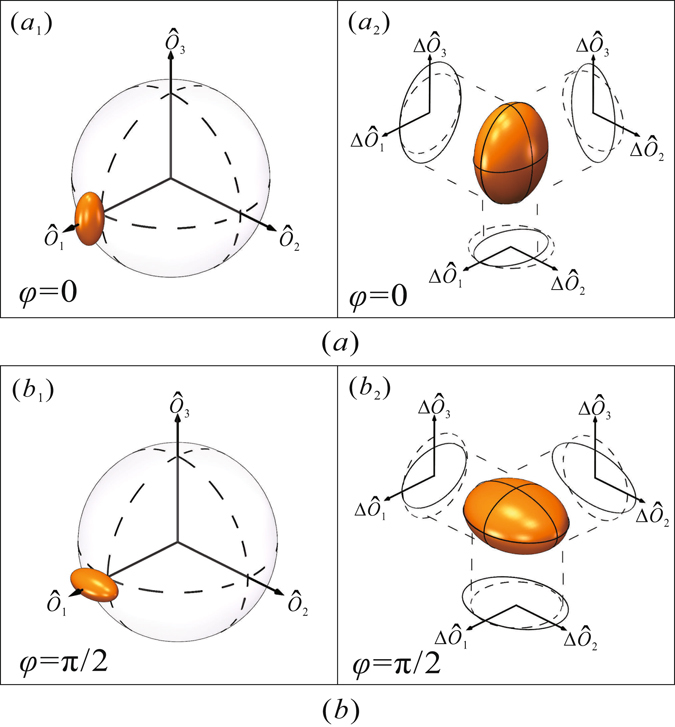
OAM squeezed states mapped onto the orbital Poincaré sphere. (**a**) orbital Poincaré sphere for *φ* = 0. (**b**) orbital Poincaré sphere for *φ* = *π*/2.

The OAM squeezed states at 5 MHz were mapped onto the orbital Poincaré sphere (Fig. 7); (a) is the orbital Poincaré sphere for *φ* = 0, (*b*) is the orbital Poincaré sphere for *φ* = *π*/2, (*a*_1_) and (*b*_1_) show positions and forms of the OAM squeezed states for *φ* = 0 and *φ* = *π*/2. As the HG_10_ mode is a bright coherent state, the OAM states are therefore positioned on the positive part of the ${\hat{O}}_{1}$ axis. (*a*_2_) shows the sphere for the quantum noise of the OAM squeezed state with *φ* = 0. Here ${\rm{\Delta }}{\hat{O}}_{1}$ is SNL, ${\rm{\Delta }}{\hat{O}}_{2}$ is squeezed, and ${\rm{\Delta }}{\hat{O}}_{3}$ is anti-squeezed; hence it is pancake shaped. Similarly (*b*_2_) shows the sphere for the state with *φ* = *π*/2. Note ${\rm{\Delta }}{\hat{O}}_{1}$ is still SNL, with ${\rm{\Delta }}{\hat{O}}_{2}$ anti-squeezed and ${\rm{\Delta }}{\hat{O}}_{3}$ squeezed, and therefore also pancake shaped. The experimental results agree with Eq. (3) well.

## Conclusion

The CV OAM squeezed states have great potential in high-dimensional quantum information processing, super-resolution quantum imaging, quantum precise measurement, and quantum storage. We demonstrated experimentally a new measurement scheme for the Stokes operators of the first-order OAM squeezed state. The OAM squeezed states are generated by coupling a HG_01_ squeezed state with a bright coherent HG_10_ mode on a 98/2 beam splitter. With the scheme, we measured the squeezing of the Stokes operators. The experiment demonstrates that the scheme is effective and efficient. The CV OAM states with the detection scheme is promising for applications in nonlocal quantum information, and the scheme may be extended to high-order CV OAM states for high-dimensional quantum information processing.
